# Spatial and temporal distribution and ecological risk assessment of typical antibiotics in natural and wastewater of Jinjiang River Basin

**DOI:** 10.1371/journal.pone.0310865

**Published:** 2024-11-14

**Authors:** Meng Wang, Jiale Li, Yongkang Zhou, Wenjia Zhou, Shuai Huang

**Affiliations:** 1 School of Geological Engineering, Institute of Disaster Prevention, Sanhe, China; 2 Hebei Key Laboratory of Earthquake Disaster Prevention and Risk Assessment, China; 3 School of Water Resources and Environmental Engineering, East China Institute of Technology, China; VIT University, INDIA

## Abstract

Antibiotics are widely used in human medical, livestock, and aquaculture fields. Most antibiotics are water-soluble and cannot be fully absorbed by humans or animals. If feces or wastewater containing antibiotics are improperly treated or discharged directly into surface water or groundwater, it will undoubtedly have an impact on aquatic organisms. The Ganjiang River is the largest river in Jiangxi Province and the largest tributary of Poyang Lake Basin. Jinjiang River, a tributary of Ganjiang River, is a typical livestock and poultry breeding area in the Poyang Lake Basin, along which many townships and counties are distributed. Gao’an and Shanggao counties are important agricultural and animal husbandry production areas in Jiangxi Province. In this paper, automatic solid phase extraction-ultra high performance liquid chromatography-mass spectrometry (SPE-UPLC-MS/MS) technology was used to simultaneously detect 27 antibiotics in 5 categories of macrolides, tetracyclines, quinolones, nitroimidazoles and sulfonamides in water. Based on this method, the concentrations and distributions of these antibiotics were analyzed. Ecological risk assessment of the Jinjiang River Basin was conducted using the ecological risk quotient method, aiming to supplement antibiotic data in the Jinjiang River Basin and provide scientific basis for local ecological environment management. The research results indicate that from 2019 to 2021, two years later, there was an increase in the use of Sulfadiazine and Roxithromycin in the Jinjiang River Basin, while the usage of Ciprofloxacin and Oxytetracycline was relatively low. In 2021, out of the 27 antibiotics, 24 were detected in surface water, 20 in groundwater, and all in wastewater. Among them, Sulfamethoxazole was the most widely used antibiotic, primarily in livestock and poultry farming. Gao’an City, a key breeding area in the Jinjiang River Basin, exhibited the highest concentration of Sulfamethoxazole at 409.96 ng·L^-1^, which far exceeds other antibiotics and warrants significant attention. A comparison of surface water concentrations between the Jinjiang River and 12 other regions revealed higher overall pollution levels of Roxithromycin and Sulfamethoxazole. Furthermore, according to the ecological risk assessment results, only Sulfamethoxazole poses a moderate risk to aquatic organisms.

## Introduction

Antibiotics are chemical substances produced by organisms in life activities, which can resist pathogens and affect the growth and development of other cells. They are widely used in human medicine, animal husbandry and aquaculture because of their functions of treating and preventing various bacterial and pathogenic microbial infections [1, 2].The world ’s consumption of antibiotics ranges from 100,000 to 200,000 tons per year, of which approximately 50% is spent on veterinary medicine and as growth promoters [3]. Veterinary antibiotics are widely used in animal therapeutic agents and feed additives worldwide. The main role of veterinary antibiotics is to prevent animal diseases and promote animal growth. Most veterinary antibiotics are water-soluble and cannot be completely absorbed by animals. Antibiotics that are not absorbed by animals will be excreted into the water and soil environment in the form of active or inactive metabolites with the feces and urine of animals. If these antibiotic-containing manure and wastewater are even discharged directly into surface water and groundwater without treatment, it will undoubtedly affect aquatic organisms. For example, oxytetracycline, doxycycline and azithromycin inhibit the synthesis of proteins in microorganisms and show great toxic effects on algae [4, 5]. In addition, trace amounts of antibiotics present in the water and soil environment can cause bacterial resistance, which is not conducive to the treatment of diseases and is harmful to human health. Over the past few decades, the number of human pathogenic bacteria resistant to one or more antibiotics has increased dramatically worldwide. In the United States, more than 2 million people are infected with antibiotic-resistant bacteria each year, and more than 20,000 of them die from these bacterial infections [6, 7].

Ganjiang River is the largest river in Jiangxi Province and the largest tributary of Poyang Lake Basin. The Jinjiang River, located in the northwest corner of Jiangxi Province, is a tributary of the Ganjiang River in the Yangtze River system. It originates from the eastern foothills of the Mufu Mountains at the border of Jiangxi and Hunan provinces, flows eastward through Wanzai County, Shanggao County, and Gao’an City, and converges with the Ganjiang River in the jurisdiction of Houtian Town, Xinjian District. The basin area is 7,886 square kilometers, with an average annual precipitation of 1,617.5 mm. The Jinjiang River Basin belongs to the southwest basin of Poyang Lake and is a typical agricultural and poultry farming area in Jiangxi Province. There are numerous breeding farms in the basin, and Gao’an, located along the river, is renowned as the "agricultural county," serving as a national pig export market and one of the top ten livestock cities in the province. In 2021, Shanggao County was approved as a demonstration area for aquaculture and ecological aquaculture. Antibiotics were detected in the Jinjiang River in 2019, with 20 types found in the river and 15 in groundwater. Wanzai, Shanggao, and Gao’an are the main residential areas in the Jinjiang River Basin, with most of the population living in rural areas and towns. Their production and domestic water mainly come from rivers and lakes within the basin, making water resources crucial for local production, agricultural development, and ecological environment.

In this study, water samples were collected from 16 sites in the Jinjiang River Basin during the high-water period of Poyang Lake in the summer months of 2021, encompassing river water, groundwater, and wastewater. The objectives of this study are as follows: (i) to elucidate the concentration characteristics and distribution patterns of 27 antibiotics belonging to macrolides, tetracyclines, nitroimidazoles, quinolones, and sulfonamides in 2021; (ii) to analyze the changes in concentration and spatial-temporal distribution of the 27 antibiotics two years later, comparing data from 2019 [8] and 2021; (iii) to compare the 2021 Jinjiang River data with antibiotic concentration data from other basins; (iv) to assess the ecological risks of multiple antibiotics to three aquatic organisms. Through these studies, it aims to provide scientific basis for antibiotic pollution prevention and control, as well as water environment management in the Jinjiang area.

## Materials and methods

### Study area

**Fig 1:**
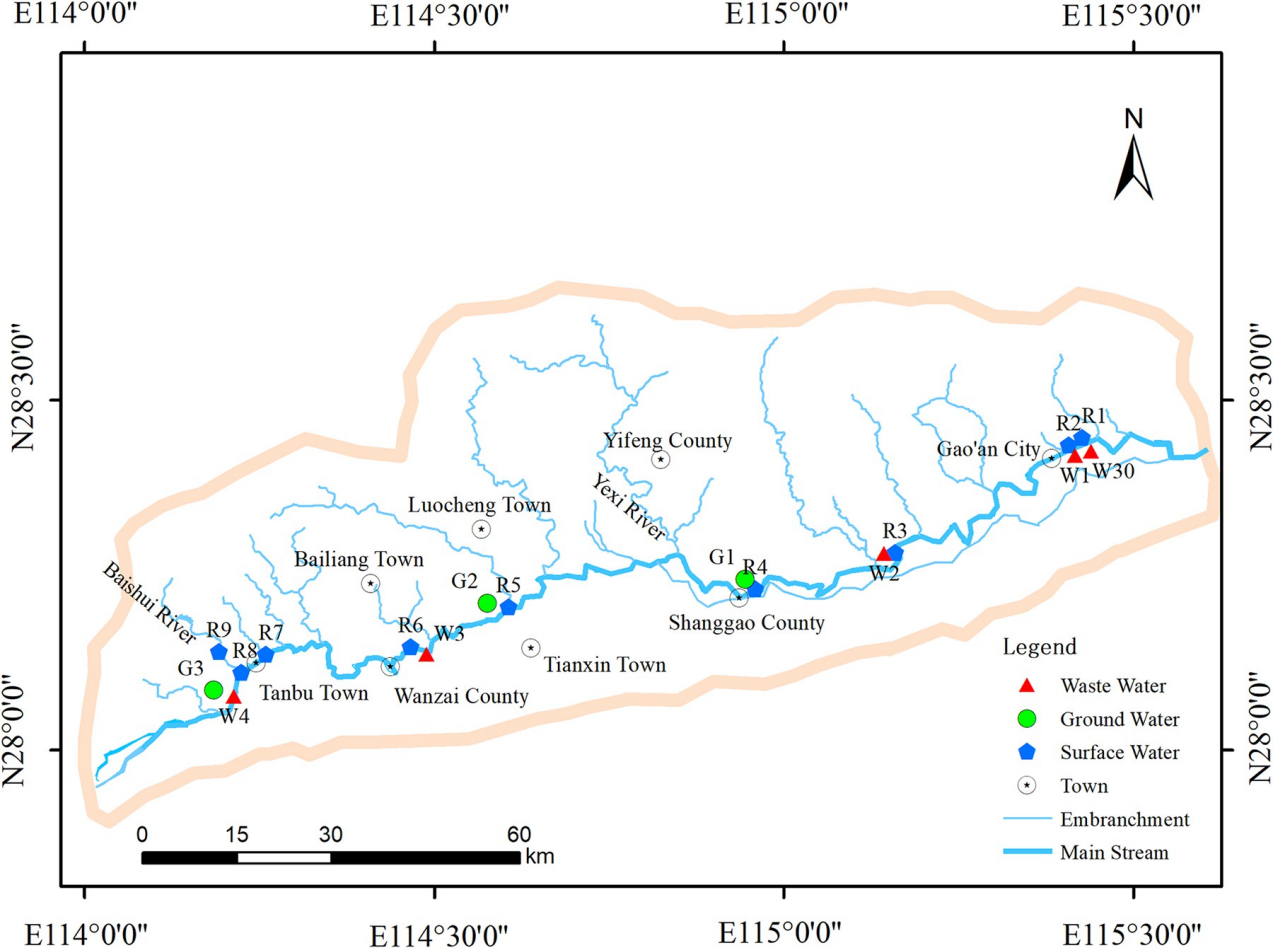
Jinjiang 2019 and 2021 sampling point map. (Basemap courtesy of Esri. Esri reserves the right to grant permission for any other use of the Image).

The sampling locations for water samples in 2019 and 2021 are shown in Fig 1 and Table 1. There were slight differences in the sampling points between 2019 and 2021. The sampling point W3 for wastewater was found to be abandoned in 2021. W30 is a sampling point added in 2021 for the domestic sewage treatment plant downstream of R2.

**Table 1:** Collection of water samples in Jinjiang River in summer months of 2019 and 2021.

Number	Acquisition time	Type	Geographical position
R1	2019/2021	River water	Upstream of Jingzhou Bridge
W30	2021	Wastewater	Sewage outlet of Jingzhou Bridge
W1	2019/2021	Wastewater	Sewage outlet of industrial wastewater treatment plant
R2	2019/2021	River water	Downstream of Jingzhou Bridge
W2	2019/2021	River water	Reservoir beside a pig farm in Shanggao county
R3	2019/2021	River water	Downstream of a pig farm in Shanggao county
R4	2019/2021	River water	Upstream of Jinjiang Bridge in Shanggao county
G1	2019/2021	Ground water	Villager’s home well in Ao Bai village
R5	2019/2021	River water	Upstream of Duzhen Dam in Jingjiang town
G2	2019/2021	Ground water	Drinking water pressure wells for residents in Zhendu Town
R6	2019/2021	River water	Under Dingtian Bridge west of Wanzai Toll Station
R7	2019/2021	River water	Baishui meets Jinjiang at the upper reaches of Lion Bridge.
R8	2019/2021	River water	Downstream of Jinjiang Lion Bridge
R9	2019/2021	River water	Baishuihe generator bypass canal
G3	2019/2021	Ground water	Shanyaoshuijing, Tanbu Village, Tanbu Town
W3	2019	Wastewater	West of Wanzai toll gate
W4	2019/2021	Wastewater	Pig farm livestock sewage pool

### Sample collection

Each sampling point collected 1 L of water samples with brown sample bottles (Shuniu glassware). Add 50 mL of methanol and 100~1000 μL of 4.6 mol/L dilute sulfuric acid solution (prepared using 98% concentrated sulfuric acid with a water to concentrated sulfuric acid volume ratio of 75:25) to each sampling point until the pH of the water sample is adjusted to around 3. Fill the bottles to the top without air bubbles, seal them, and store them in the dark at low temperature until they are transported back to the laboratory. Follow the storage conditions specified in "Technical Guidelines for the Preservation and Management of Water Quality Samples" (HJ493-2009) to ensure that testing is completed within 24 hours. The role of adding sulfuric acid is to improve the extraction efficiency of antibiotics, while the function of adding methanol is to inhibit bacteria and prevent the degradation of antibiotics by microorganisms in water samples.

### Instrument and equipment

Instruments include: Agilent 1290–6460 liquid chromatography-mass spectrometry (Agilent Technologies Co., Ltd.), UVS-3 vortex mixer (Beijing Yousheng United Technology Co., Ltd.), AR224CN electronic balance (Ohaus Instruments Co., Ltd.), SCAA-SF1000 solvent filter (Shanghai Anpu Experimental Technology Co., Ltd.), GM-0.33A diaphragm vacuum pump (Tianjin Jinteng Experimental Equipment Co., Ltd.) and Direct-Q ® 5UV ultrapure water machine (Merck, Germany). The information of antibiotics and recovery indicators used in the experiment is as follows. The drugs were imported from Dr.Ehrenstorfer, Germany (Table 2).

**Table 2:** Antibiotic standard and recovery indicator information table.

Class	Abbreviation	Name	Molecular formula	CAS
MLs	RTM	Roxithromycin	C_41_H_76_N_2_O_15_	80214-83-1
	ERY	Erythromycin	C_37_H_67_NO_13_	114-07-8
	AZM	Azithromycin	C_38_H_72_N_2_O_12_·2H_2_O	83905-01-5
	CTM	Clarthromycin	C_38_H_69_NO_13_	81103-11-9
TCs	CTC	Chlortetracycline	C_22_H_23_CIN_2_O_8_·HCl	64-72-2
	OTC	Oxytetracycline	C_22_H_24_N_2_O_9_·HCl	2058-46-0
	DOC	Doxycycline	C_24_H_30_N_2_O_9_·HCl	24390-14-5
	TC	Tetracycline	C_22_H_24_N_2_O_8_·HCl	64-75-5
FQs	ENR	Enrofloxacin	C_19_H_22_FN_3_O_3_	93106-60-6
	CIP	Ciprofloxacin	C_17_H_18_FN_3_O_3_.HCL	93107-08-5
	OFL	Ofloxacin	C_18_H_2_OFN_3_O_4_	82419-36-1
	ENO	Enoxacin	C_15_H_17_FN_4_O_3_	74011-58-8
	NOR	Norfloxacin	C_16_H_18_FN_3_O_3_	70458-96-7
	SPA	Sparfloxacin	C_19_H_22_F_2_N_4_O_3_	110871-868-8
	GAT	Gatifloxacin	C_19_H_22_FN_3_O_4_	112811-59-3
	FLE	Fleroxacin	C_17_H_18_F_3_N_3_O_3_	79660-72-3
	LOM	Lomefloxacin	C_17_H_19_F_2_N_3_O_3_	98079-52-8
NDs	MDZ	Metronidazole	C_6_H_9_N_3_O_3_	443-48-1
	DMZ	Dimetridazole	C_5_H_7_N_3_O_2_	551-92-8
SAs	SDZ	Sulfadiazine	C_10_H_10_N_4_O_2_S	68-35-9
	SMX	Sulfamethoxazole	C_10_H_11_N_3_O_3_S	723-46-6
	STZ	Sulfathiazole	C_9_H_9_N_3_O_2_S_2_	72-14-0
	SQX	Sulfaquinoxaline	C_14_H_12_N_4_O_2_S	59-40-5
	SFM	Sulfameter	C_11_H_12_N_4_O_3_S	651-06-9
	SMZ	Sulfamethazine	C_12_H_14_N_4_O_2_S	57-68-1
	STP	Sulfamethoxypyridazine	C_11_H_12_N_4_O_3_S	80-35-3
	SPD	Sulfapyridine	C_11_H_11_N_3_O_2_S	144-83-2
Deuterated indicator	ERY-^13^C,D_3_	Erythromycin-^13^C,D_3_	C_36_^13^CH_64_D_3_NO_13_	959119-26-7
	DTC	Demeclocycline	C_21_H_21_ClN_2_O_8_	64-73-3
	CIP-D8	Ciprofloxacin-D8	C_17_H_10_D_8_FN_3_O_3_	1216659–5
	SMR-D4	Sulfamerazine-D4	C_11_H_8_D_4_N_4_O_2_S	1020719–8

Note: The antibiotic names mentioned in the subsequent text will be referred to using the corresponding abbreviations in this table.

### Sample preparation and detection

The pre-treatment method of water samples is solid-phase extraction. Meanwhile, the parameters and consumables involved in the pre-treatment process are optimized to establish an efficient pre-treatment model for water samples. The following is the specific experimental procedure:

- The 1L water sample was filtered using a 1000 mL suction filter, and the 0.7μm GF / F filter membrane (whatman) was selected. After the filtration, the filtered water sample was poured into a washed brown bottle and 0.5 g Na_2_EDTA was added and stirred evenly.
- For the same batch of samples, set up two blanks and two quality controls (QC). Add relevant substances recovery indicators (4 mixed standards) into each water sample in brown bottles (default 1 L) at a concentration of 100 ng/L after addition. For QC, add 27 standard substances and recovery indicators.
- Set up the automated solid-phase extraction instrument program (Heffic S6). Activate the small column with 10 mL of methanol and 10 mL of ultrapure water, then pass the mixed water sample through the activated solid-phase extraction column at a flow rate of 8 mL/min. After extraction, use a vacuum pump to dry until there are no liquid drops at the tip of the tube.
- 12 mL 0.1% formic acid methanol was used to elute the extraction column, and the liquid was dropped into 30 mL test tube.
- Evaporate the extract to near dryness with nitrogen, dissolve it in 1 mL of methanol-water solution, filter through a 0.22 μm organic filter membrane, and evaporate to near dryness again with 100 μL of nitrogen. Finally, resuspend the extract in 100 μL of initial mobile phase solution, and place the final extract in a 2 mL brown bottle with a liner for analysis.
- Agilent 1290–6460 was used as the detection instrument, and the chromatographic column was ZORBAX Eclipse Plus C18 (RRHD) 1.8μm. The column oven temperature was 25°C, and the sample injection volume was 10 μL. The mobile phase mixing gradient is shown in S1 Table. Mass spectrometry was performed using a positive ion mode ESI electrospray ion source, capillary voltage of 3500 V, corona current of 4 μA, MRM detection mode. The drying gas temperature and the atomization chamber temperature were set to 325°C, the drying gas flow rate was 6 L/min, and the sprayer pressure was 40 psi. The MRM acquisition parameters of 27 target antibiotics deuterated indicators and the internal standard are detailed in S2 Table.

### Quality assurance and quality control

The standard curve concentration gradient was set to 0.1,0.2,0.5,1,2,5,10,20,50,100,200,500 μg·L^-1^, which could basically cover the concentration range of antibiotics in environmental water. The standard solution contains 27 standards and 4 deuterated indicators. The internal standard Simeton was added to each standard solution and the sample after pretreatment, so that the internal standard concentration detected by the machine was 100 μg·L^-1^. The standard curve exhibits good linearity (R^2^ > 0.995), as shown in S3 Table. The limit of detection was 0.01~0.44 ng·L^-1^, and the limit of quantitation was 0.03~1.36 ng·L^-1^.

Blank water samples were prepared with ultrapure water, and the standard concentration was considered at low, medium and high levels. The mixed standard solution of 10,50,100 ng·L^-1^ antibiotics was added to the blank water samples, and the recovery rate and relative standard deviation of each spiked concentration were calculated. The results are shown in S4 Table. The recoveries of antibiotics are between 28.38% and 112.58%, and the relative standard deviations are not more than 11% (n = 3). Select three surface water samples as matrices, spike them at low, medium, and high levels with the same method, and conduct analysis. The recovery rates of antibiotic matrix spiking range from 26.22% to 280.02%, with relative standard deviations all below 12%.

### Ecological risk assessment method

In this paper, the ecological risk assessment method of antibiotics is carried out according to the EU technical guidance document [9]. The specific calculation formula is as follows:

$$RQ_{S}=MEC/PNEC$$ (1)

PNEC=L(E)C50/AForPNEC=NOEC/AF (2)

In the formula, RQ_S_ is the risk quotient, MEC is the measured concentration of pollutants (ng·L^-1^), PNEC is the predicted no effect concentration (ng·L^-1^), L (E) C50 is the median lethal concentration and the median maximum effect concentration, NOEC is the no observed effect concentration, AF is the assessment factor, and the value of PNEC is L (E) C50 or NOEC divided by AF. The AF value is selected according to the biological toxicity data in the technical guidance of the European Union [9]. When the toxicological data is selected as L (E) C50, the AF value is 1000; when the NOEC was selected as the toxicological data, the AF value was 100. The toxicological data for each antibiotic were retrieved from the ECOSAR v2.2 toxicity database, which conducted toxicity tests on aquatic organisms, including algae, invertebrates, and fish. Given that antibiotics are typically present in trace amounts in aquatic environments, meaning their concentration s in water are insufficient to produce unacceptable effects, the most sensitive toxicity test data, namely NOEC, was chosen for further analysis. Additional details can be found in Table 3.

**Table 3:** Toxicity data of antibiotics for aquatic organisms calculated by ECOSAR.

Class of antibiotics	Abbreviation	Green algae (mg/L)	Daphnid (mg/L)	Fish (mg/L)	Type	Assessment factor
MLs	RTM	1.65	0.60	2.30	NOEC	100
	ERY	2.20	0.75	3.60	NOEC	100
	AZM	0.69	0.29	0.82	NOEC	100
TCs	CTC	0.63	4.17	20.20	NOEC	100
	OTC	10.3	2.84	4.48	NOEC	100
	DOC	0.80	7.62	45.3	NOEC	100
	TC	3.28	1.25	1.29	NOEC	100
FQs	ENR	167	35.8	454	NOEC	100
	CIP	455	81.3	1550	NOEC	100
	OFL	645	114	2460	NOEC	100
	ENO	608	103	2220	NOEC	100
	NOR	703	116	2650	NOEC	100
NDs	MDZ	2.70	3.08	0.95	NOEC	100
	DMZ	0.87	0.66	0.29	NOEC	100
SAs	SDZ	29	0.1	23.8	NOEC	100
	SMX	5	0.068	11.1	NOEC	100
	SMZ	8.88	0.065	3.26	NOEC	100

At the same time, according to the RQ classification method proposed by Hernand [10], RQ can be divided into four categories: RQ < 0.01 is risk-free, 0.01 ≤ RQ < 0.1 is low risk, 0.1 ≤ RQ < 1 is medium risk, and RQ ≥1 is high risk.

## Results and discussion

### Concentration level of antibiotics in Jinjiang River Basin

**Fig 2:**
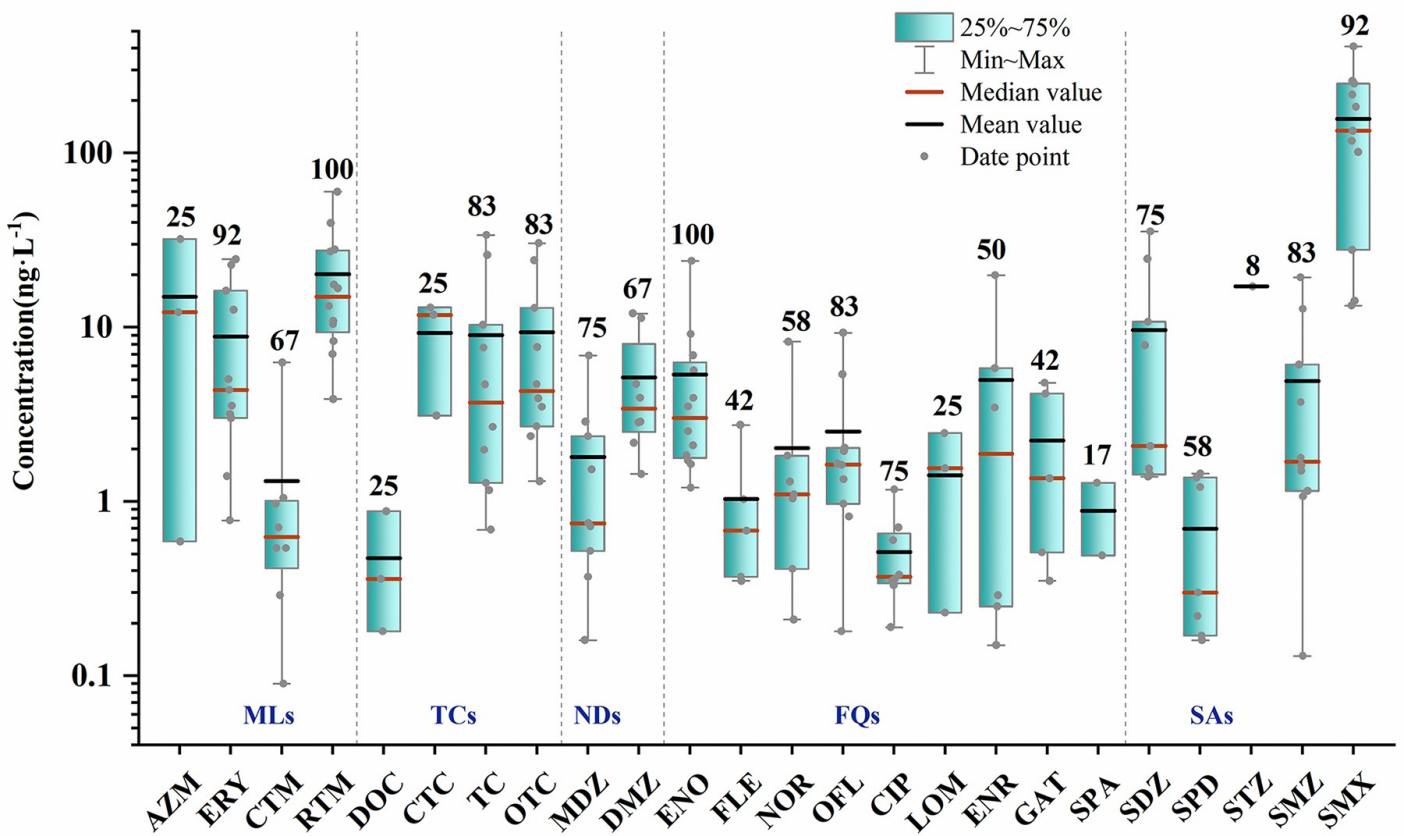
Boxplot of antibiotic concentrations in surface water and groundwater in 2021 (N = 12). (The numbers above the boxes represent the detection rate of each antibiotic %).

In 2021, 24 antibiotics were detected in surface water, while 20 antibiotics were detected in groundwater. All 27 antibiotics were detected at four wastewater sampling points (Table 4). Two antibiotics, RTM and ENO, had a detection rate of 100% in both surface water and groundwater (Fig 2). The highest detected concentration was for SMX, reaching 409.96 ng·L^-1^, followed by RTM at a maximum of 59.99 ng·L^-1^. Individual antibiotic concentrations in groundwater did not exceed 30 ng·L^-1^, with TC being the highest at 26.04 ng·L^-1^, similar to surface water characteristics. The next highest was ENO at 24.08 ng·L^-1^, with lower concentrations detected for other antibiotics.

**Table 4:** Concentration characteristics of 16 sampling points in Jinjiang River Basin.

Class of antibiotics		Surface water (*n* = 9)		Groundwater (*n* = 3)		Wastewater (*n* = 4)	
		Concentration /ng·L^-1^	Mean value /ng·L^-1^	Concentration /ng·L^-1^	Mean value /ng·L^-1^	Concentration /ng·L^-1^	Mean value /ng·L^-1^
MLs	AZM	ND~32.15	3.64	ND~12.20	4.07	ND~14.05	4.37
	ERY	1.40~24.61	10.27	ND~4.37	1.72	2.92~10.61	6.75
	CTM	ND~1.05	0.47	ND~6.29	2.10	ND~7.36	2.25
	RTM	3.88~59.99	22.52	10.42~16.75	13.48	ND~53.58	32.88
	∑MLs	8.41~83.90	36.89	11.19~39.61	21.36	8.82~83.05	46.24
TCs	DOC	ND~0.88	0.16	ND	ND	ND~1838.68	459.67
	CTC	ND~13.03	2.76	ND~3.11	1.04	ND~27.18	6.92
	TC	ND ~33.90	7.01	ND~26.04	9.11	2.37~35.17	11.67
	OTC	ND~30.43	9.75	ND~4.72	2.01	3.69~1826.29	459.57
	∑TCs	3.06~58.11	19.68	ND~33.87	12.16	6.6~3727.33	937.82
FQs	ENO	1.20~9.16	3.50	1.84~24.08	10.95	ND~70.94	19.45
	FLE	ND~2.75	0.46	ND~1.03	0.34	ND~34.71	8.87
	NOR	ND~8.27	1.35	ND~1.83	0.68	0.27~156.44	40.98
	OFL	0.18~9.31	2.66	ND~1.34	0.45	0.28~250.41	84.09
	CIP	ND~1.17	0.35	ND~0.60	0.32	0.22~316.76	80.87
	LOM	ND~2.47	0.30	ND~1.55	0.52	ND~1.54	0.38
	ENR	ND~19.96	2.68	ND~5.84	1.95	ND~1318.86	331.67
	GAT	ND~4.81	0.78	ND~4.16	1.39	ND~137.22	35.44
	SPA	ND~0.49	0.05	ND~1.28	0.43	ND~0.41	0.10
	∑FQs	4.49~52.69	12.12	2.40~24.56	17.01	3.56~2285.73	601.86
NDs	MDZ	ND~6.89	1.72	ND~0.72	0.24	0.11~2.56	1.28
	DMZ	ND~12.03	4.35	ND~2.17	0.72	2.06~113.11	40.84
	∑NDs	ND~14.18	6.07	ND~2.17	0.97	2.18~115.42	42.12
SAs	SDZ	1.39~35.48	9.63	ND	ND	ND~169.2	42.34
	SPD	ND~1.45	0.54	ND	ND	ND~2.9	0.92
	STZ	ND~17.19	1.91	ND	ND	ND~32.18	8.04
	SMZ	1.07~19.35	5.45	ND~0.13	0.04	0.26~9.48	4.17
	SFM	ND	ND	ND	ND	ND~9.79	2.45
	STP	ND	ND	ND	ND	ND~9.41	2.35
	SMX	27.85~409.96	188.95	ND~14.21	9.18	ND~62.24	33.65
	SQX	ND	ND	ND	ND	ND~1.12	0.28
	∑SAs	31.00~431.01	206.49	ND~14.21	9.23	30.33~231.17	94.21

Note: ND = not detected

**Fig 3:**
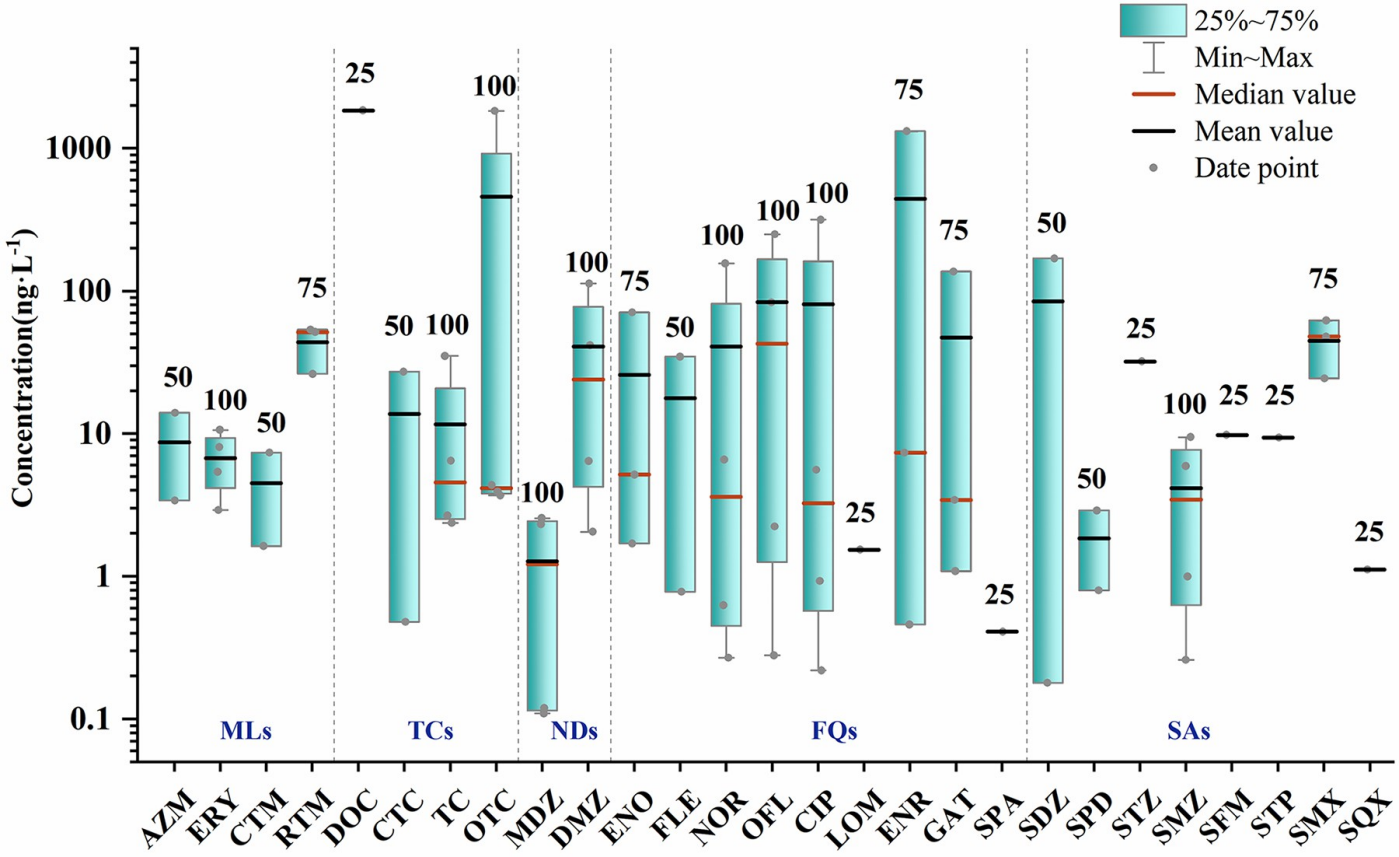
Boxplot of antibiotic concentrations in wastewater in 2021 (N = 4). (The numbers above the boxes represent the detection rate of each antibiotic %).

Wastewater includes pig farm effluent, domestic sewage, and industrial wastewater. Nine antibiotics had recovery rates exceeding 100%, with STP, SFM, and SQX detected only at pig farm effluent sampling points (Fig 3). Pig farm effluent collected from a pig farm on the hill had the highest antibiotic detection concentrations. Tetracyclines and quinolones were primarily used for poultry and livestock feeding. The detected concentrations of DOC, OTC and ENR were 1838.68 ng·L^-1^, 1826.29 ng·L^-1^ and 1318.86 ng·L^-1^ respectively, followed by NOR, OFL, CIP and GAT, all greater than 100 ng·L^-1^, which was much higher than that in surface water and groundwater.

### Spatial distribution of antibiotic concentration in Jinjiang

**Fig 4:**
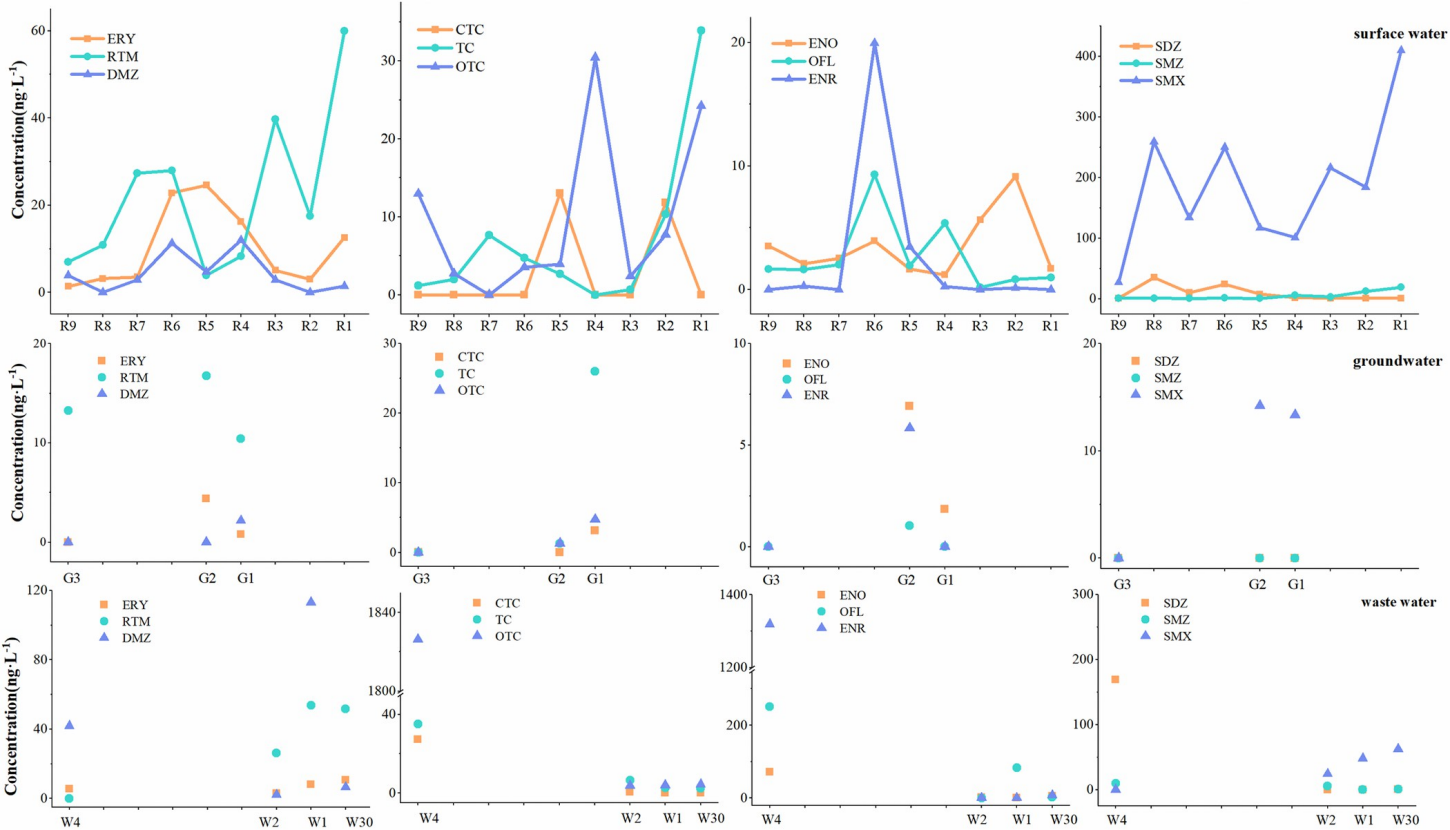
Concentrations of various antibiotics in Jinjiang River.

The sampling points of surface water, groundwater, and wastewater were plotted separately in the direction from the river source to the estuary, and the positions of groundwater and wastewater in Fig 4 corresponded one-to-one with the surface water. R9 is the Baishui River, where the overall antibiotic concentration is relatively low, and there is basically no pollution. R8 was collected from the Jinjiang River, with a high concentration of SMX. SMX was also detected at two downstream groundwater sampling sites. From the river source to the estuary, the Baishui River flows into the Jinjiang River. Due to the dilution effect of the river inflow, the overall antibiotic concentration from R8 to R7 shows a decreasing trend, and the SMX concentration decreases from 258.88 ng·L^-1^ to 134.17 ng·L^-1^. R6 is located near the residential area of Wanzai County, where human activities are frequent, and there are medical discharges and animal husbandry activities, resulting in an increase in the concentration of various antibiotics. There are breeding activities near R3, and the increase in concentrations of sulfonamides and macrolides antibiotics may be related to them. R2 and R1 are located in the residential areas of Gao’an City, where human activities are frequent, and high concentrations of SMX are detected in the river, reaching a maximum of 409.96 ng·L^-1^ at R1.

No high-concentration sulfamethoxazole was found during the detection at the discharge outlets of an industrial wastewater and domestic sewage treatment plant nearby, so there might be other pollution sources. W4 is the wastewater collection area of a pig farm on the hill. The detected concentration of tetracycline antibiotics exceeded 1000 ng·L^-1^. Since the wastewater was stored in a closed environment and did not flow, it did not affect the groundwater and surface water downstream. Additionally, while high levels of SMX were detected in surface water, it was not detected in pig farming wastewater, indicating that the pig farm might not have used SMX as an additive in pig farming.

### Changes in antibiotic concentration over time

**Fig 5:**
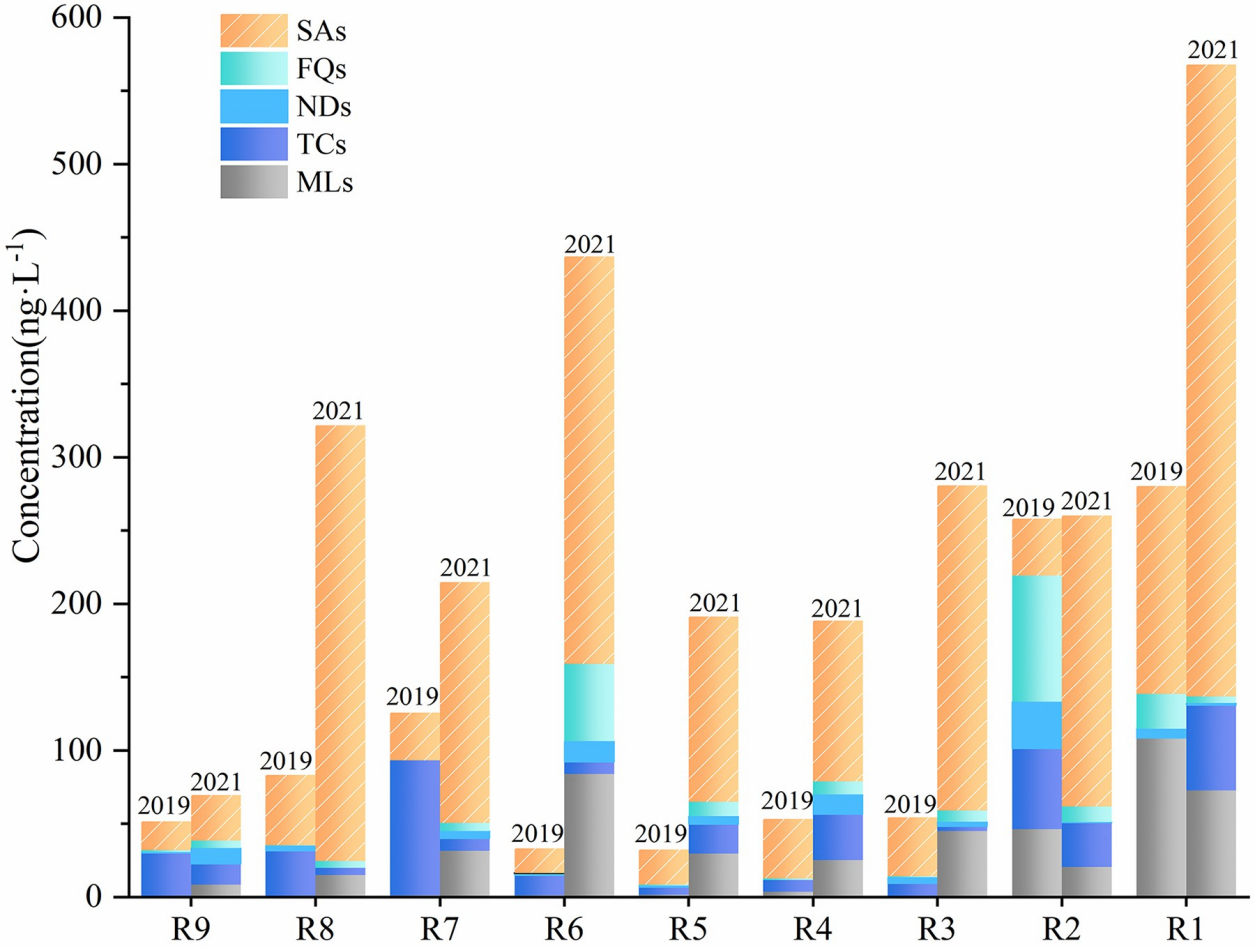
Concentration changes of five types of antibiotics along the Jinjiang river flow.

Comparing the antibiotic concentrations at nine surface water sampling points from the river source to the estuary in 2019 and 2021 (Fig 5), it was found that the total antibiotic concentration increased. Among them, SMX has the highest increase, and its concentration accounts for 21% and 67% of the total antibiotic concentrations at the sampling points in 2019 and 2021. The SMX concentration at all sampling sites in 2021 was 8.3 times higher than that in 2019. In 2019, most of the sampling points of SMX were below 50 ng·L^-1^, and then generally around 200 ng·L^-1^ in 2021, and each sampling point had different degrees of increase. SMX can effectively inhibit the growth and reproduction of bacteria, and has a good effect on Staphylococcus and Escherichia coli in the respiratory tract. Therefore, it is widely used in livestock and poultry for urinary tract infection, respiratory tract infection and other diseases [11]. SMX has strong water solubility and mainly exists in the water environment. It is highly stable in water chemistry, not easy to degrade and easy to move in water. It is difficult to remove it by hydrolysis or biodegradation [12]. The escalating concentration of SMX suggests a widespread utilization of this compound in the Jinjiang River Basin. SMX has been globally detected, and when comparing the maximum concentrations recorded in rivers, the United States (220 ng·L^-1^) [13] < Spain Catalonia (312.2 ng·L^-1^) [14]< this study (409.96 ng·L^-1^) < Germany Baden (410 ng·L^-1^) [15], indicating that the high pollution level of SMX should be paid attention to. Another antibiotic deserving attention is RTM.the concentration of RTM in nine surface water sampling points stood at 13.96 ng·L^-1^, but it significantly escalated to 202.64 ng·L^-1^ in 2021, which was 14 times that of two years ago. Notably, these concentrations were predominantly observed in proximity to urban areas. RTM is widely used in the treatment of infectious diseases because it can fight against most bacterial and viral infections, especially respiratory infections. Because of its high production efficiency and low cost, it is often used in veterinary, treatment and prevention of diseases [16]. RTM is highly resistant to microbial biodegradation [17], so it is difficult to remove. It was reported that the average concentration of roxithromycin was 15.24 ng·L^-1^ in the groundwater of Lahore, Pakistan, while RTM was not detected in the groundwater of Xiong’an New Area [18], Jianghan Plain [19]and Barcelona, Spain [20]. It shows that there is RTM pollution in Jinjiang River Basin, and the high degree of pollution should be paid attention to.

**Fig 6:**
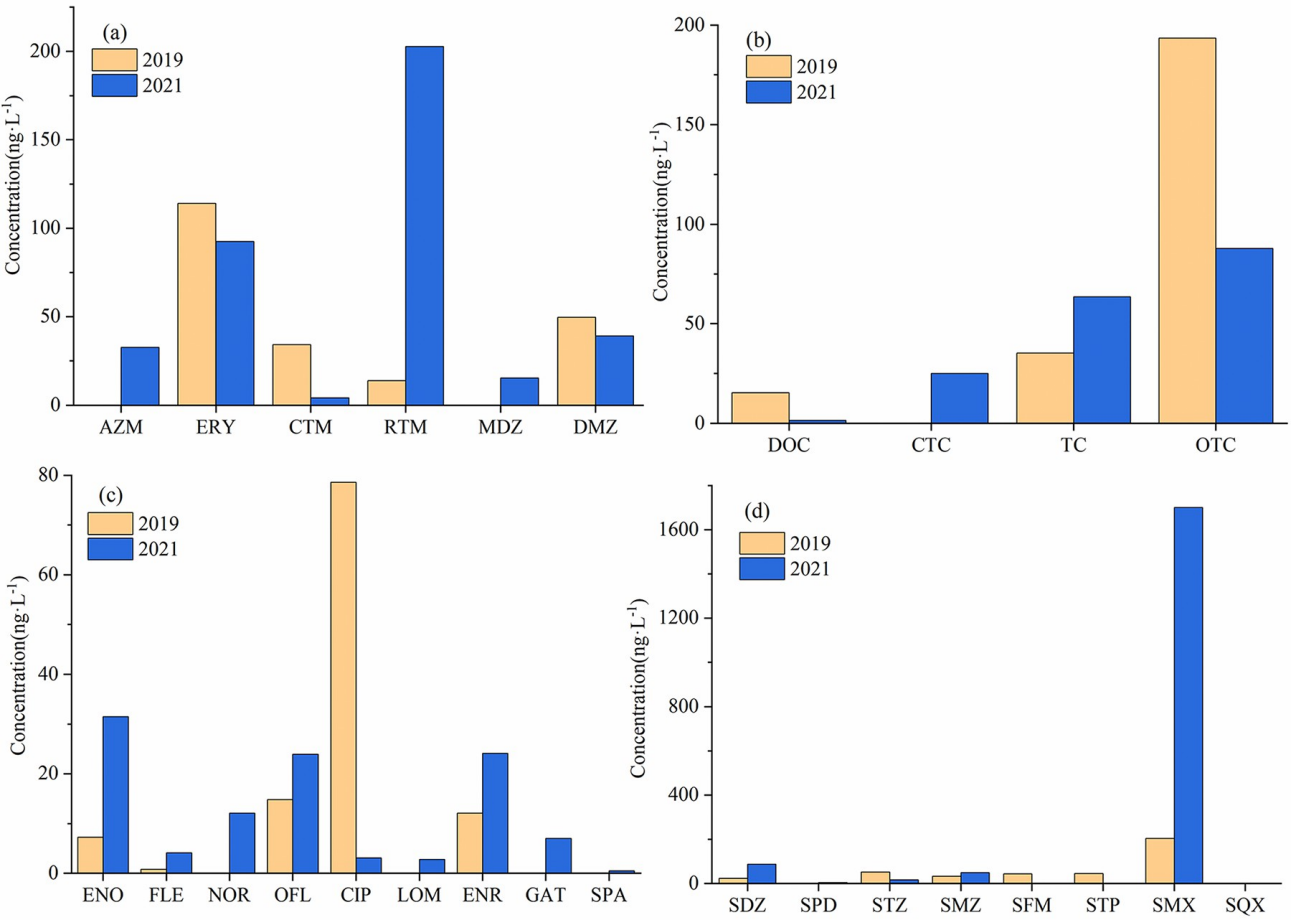
Changes in the cumulative concentration of individual antibiotics at nine surface water sampling points after two years.

Apart from the increase in the concentration of some antibiotics, there is also a decrease in the concentration of some antibiotics. As shown in Fig 6, the concentration of CIP at the nine sampling points decreased from 78.64 ng·L^-1^ in 2019 to 3.11 ng·L^-1^ in 2021, and there was almost no pollution, indicating that the demand for CIP in the Jinjiang River Basin has been reduced. The concentration of OTC in 2021 was half that of 2019, and the proportion of tetracycline antibiotics also decreased. OTC accounted for 85% and 46% of the tetracycline antibiotic concentrations in 2019 and 2021 respectively, but since OTC in sediment was not analyzed in this study, it cannot be inferred whether the usage of OTC decreased in 2019. This is related to the special properties of tetracyclines. Due to the high solid / water partition coefficient of these antibiotics, it is easy to adsorb to suspended and deposited sediments, and it is also easier to hydrolyze and photolysis [21].

### Comparative analysis of antibiotic concentration

Eleven antibiotics were chosen from a total of 27 antibiotics for comparison with antibiotics found in 12 domestic basins. These 11 antibiotics are currently in high demand for research and possess extensive research data. As shown in Table 5, there are differences in the types and levels of antibiotic pollution in different regions. When comparing the Jinjiang River with other rivers, RTM and SMX exhibit relatively high pollution levels, while the Liao River and Fen River have the highest antibiotic concentrations among all these rivers. Compared to lakes, RTM and SMX also show higher pollution levels, with Baiyangdian and the Jianghan Plain having the highest antibiotic concentrations among these lakes.

**Table 5:** Comparison of typical antibiotic pollution between Jinjiang River and other basins.

River basin (ng·L^-1^)	MLs		TCs				FQs		SAs		
	RTM	ERY	DOC	CTC	TC	OTC	ENO	ENR	SDZ	SMX	SMZ
Jinjiang river*	22.52	10.27	0.16	2.76	7.01	9.75	3.50	2.68	9.63	188.95	5.45
Yangtze river delta [22, 23]	0.9	3.9	11.3	3.6	4.2	78.3	1.0	2.8	53.6	259.6	188.9
Yellow river delta [24, 25]	10.24	2.71	-	-	18.57	15.78	121	10.06	1.82	34.4	12.9
Liao river [25]	51.4	160	0.61	10.7	3.97	56.6	56	10.12	4.51	224	28
Fen river [26]	35.42	18.54	4.76	4.10	0.47	13.27	16.92	15.42	36.82	141.89	6.2
Nansi lake [27]	5.92	4.72	12.9	ND	45.0	ND	-	-	ND	5.54	ND
Jianghan plain [28]	3.08	135.3	4.47	15.94	55.86	25.13	-	4.03	2.41	4.06	2.9
Xiaoqing river [29]	8.86	33.3	-	-	-	-	6.3	ND	9.7	134	-
Tai lake [30, 31]	ND	1139.4	0.21	2.6	1.07	0.74	-	-	2.52	116.48	34.41
Baiyangdian lake [32–35]	27.2	19.5	4.87	-	25.95	27.27	8.62	1.28	118	240	5.25
Yitong river [36]	-	-	-	-	65.88	ND	-	-	66.5	68.88	48.38
Kaiyang river [37]	27.5	45.4	ND	0.7	32.9	34.7	7.9	2.0	-	-	-
Chao lake [38, 39]	ND	7.01	1.2	1.3	1.6	0.9	-	1.5	4.5	56.1	3.2

Note:* Representing the study area; ND, not detected; ‘-’means missing data.

### Ecological risk assessment

**Fig 7:**
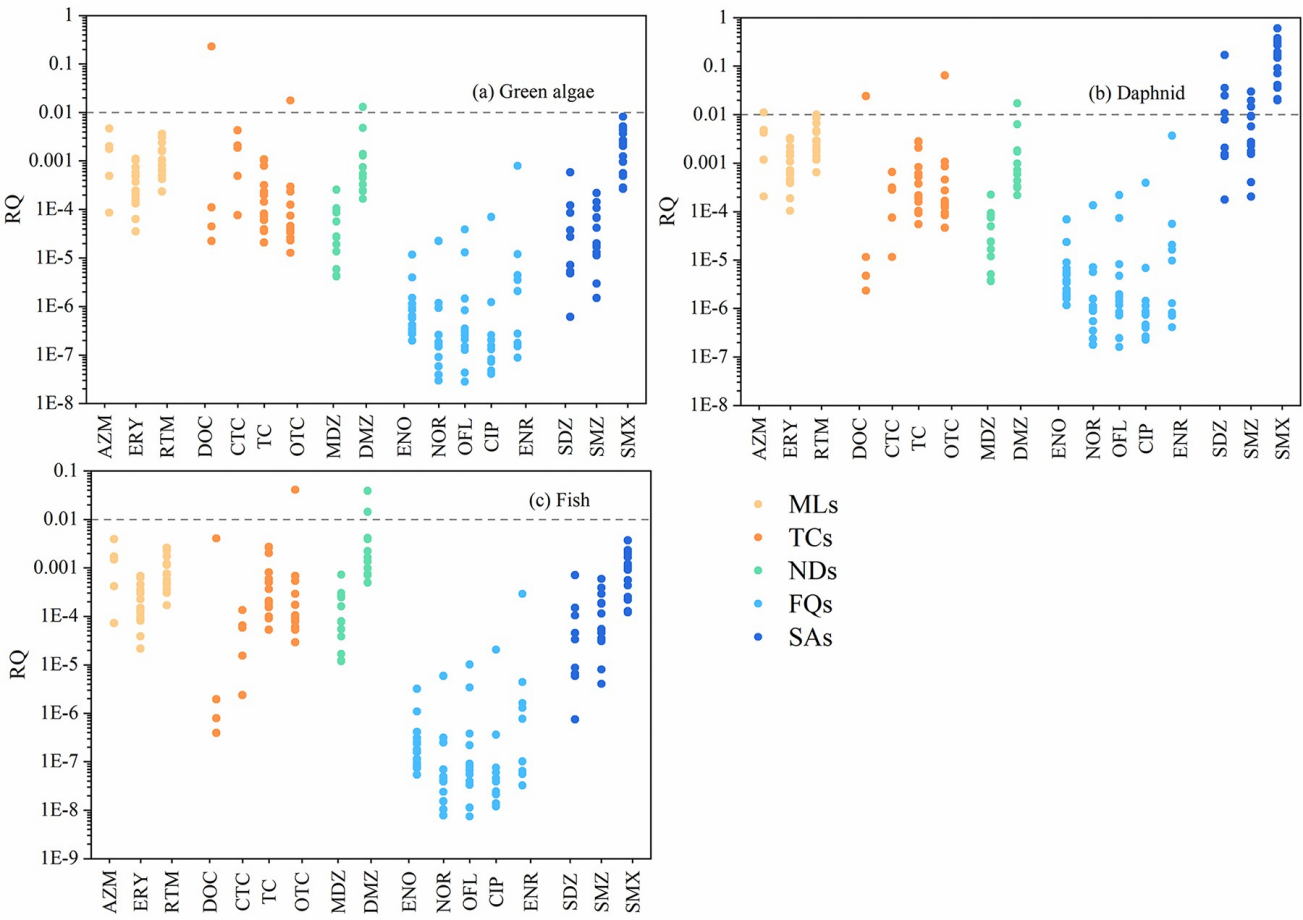
The RQ of antibiotics on three aquatic organisms at 16 sampling points. (five colors correspond to five major classes of antibiotics).

Seventeen antibiotics with high concentrations or high detection rates were selected from 27 antibiotics for ecological risk assessment, and three modes of green algae, daphnid and fish were selected for aquatic organisms. According to Table 3, the optimized risk quotient method was used to calculate the RQ values of single antibiotics for three aquatic organisms at each sampling point in Jinjiang River Basin. The calculation results are shown in Fig 7. It can be seen from the figure that only the risk quotient values of some sampling points are greater than 0.01, and the antibiotic risk quotient value of wastewater is significantly higher than that of surface water and groundwater. The concentrations of various antibiotics in surface water and groundwater have no risk for green algae and fish. SMX in surface water and groundwater may pose a medium risk to daphnid. Although the concentrations of AZM, SDZ, SMZ are not high, they are sensitive to daphnid, so they are also at low risk. The concentrations of various antibiotics in the wastewater of Jinjiang River are high, especially in pig breeding wastewater. DOC poses a medium risk to algae, and SDZ poses a medium risk to daphnid. DOC, SMZ, SMX pose a low risk to daphnid,. In addition, DMZ and OTC pose a low risk to all three aquatic organisms.

**Fig 8:**
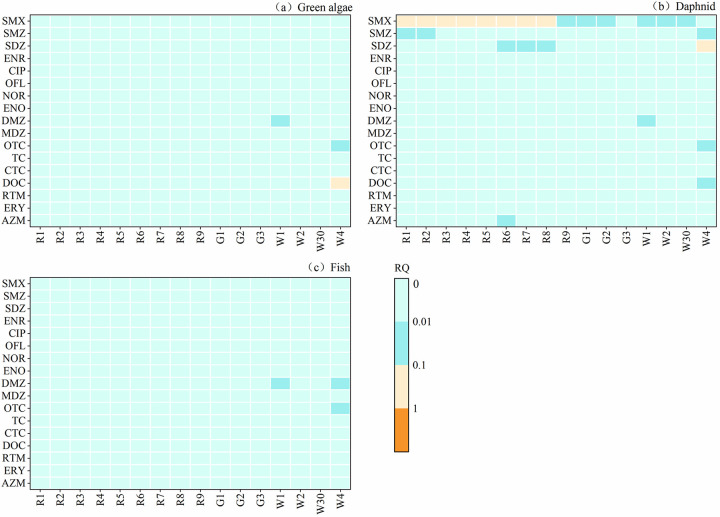
The ecological risk levels of three aquatic organisms corresponding to 16 sampling points.

The ecological risk levels of antibiotics in surface water, groundwater and wastewater in Jinjiang River Basin are shown in Fig 8. Except for sulfonamide antibiotics, surface water and groundwater have no potential ecological risks to the three aquatic organisms. SMX in all surface water sampling sites except R6 had a medium risk to daphnid, indicating that the risk level of SMX in Jinjiang River Basin was high, and the use of SMX should be properly controlled. The sensitivity of daphnid to AZM, SMZ and SDZ was also high, so some sampling points were at low risk. As can be seen from the figure, the number of antibiotic risk categories in wastewater is higher than that in surface water and groundwater, indicating that wastewater discharged from some aquaculture industries and sewage treatment plants still poses certain risks to aquatic organisms.

## Conclusion

The Jinjiang River Basin is facing antibiotic pollution, with an overall increasing trend observed in 2021 compared to 2019. In 2021, antibiotics were detected in all 16 water samples from surface water, groundwater, and wastewater. Specifically, antibiotics such as STP, SFM, and SQX were only detected in pig wastewater sampling points. Eleven antibiotics had detection rates exceeding 70%, including ERY, RTM, TC, OTC, MDZ, DMZ, ENO, OFL, CIP, SMZ, and SMX.

From a spatial distribution perspective, the concentration of sulfonamide antibiotics exhibits high levels and significant fluctuations. Notably, sulfamethoxazole concentrations are significantly higher compared to several other antibiotics, even surpassing the concentrations of the other four types of antibiotics. Specifically, in Gao’an City, the concentration of sulfonamide antibiotics peaked, with sulfamethoxazole concentrations reaching 409.96 ng·L^-1^. In terms of time, the sum of antibiotic concentrations at nine river sampling points in 2021 was 2.6 times that in 2019, with the concentrations of SMX and RTM in 2021 being 8.3 and 14.5 times those in 2019. This indicates that the use of SMX and RTM in Jinjiang River is increasing, and these antibiotics account for a large proportion of the overall antibiotic concentration. Secondly, CIP and OTC decreased significantly, with CIP falling to the level of no pollution and OTC halving in concentration. This suggests that there has been a change in antibiotic use in Jinjiang River over the past two years, with the demand and usage of CIP and OTC gradually decreasing.

After comparing with the concentrations of surface water in 12 different regions, we found that the pollution levels of RTM and SMX in the surface water of Jinjiang River were relatively high, while the pollution levels of ERY, CTC, TC, OTC, ENR, SDZ, and SMZ were moderate, which should be paid full attention to. The ecological risk of individual antibiotics at each sampling point of Jinjiang River was evaluated by using the risk quotient method. The results showed that the antibiotics posing medium risk to aquatic organisms in surface water and groundwater of Jinjiang River were SMX, while AZM, SDZ, and SMZ posed low risk. The antibiotics posing medium risk to aquatic organisms in wastewater were DOC and SDZ, while DMZ, OTC, SMX, and SMZ posed low risk.

## Supporting information

S1 Table Instrument gradient elution procedure. (DOCX)

S2 Table Mass spectrometry conditions of UPLC-MS /MS for determination of antibiotic. (DOCX)

S3 Table Limit of detection, limit of quantitation, and standard curve of antibiotics. (DOCX)

S4 Table Recoveries of antibiotics in spiked surface water and pure water samples (n = 3). (DOCX)

S1 Data (XLSX)
