# Supplementary material for: Spatial and temporal distribution and ecological risk assessment of typical antibiotics in natural and wastewater of Jinjiang River Basin
Source: PLoS One. 2024 Nov 14;19(11):e0310865. doi: 10.1371/journal.pone.0310865 (PMC11563446; doi:10.1371/journal.pone.0310865)
Supplement: S1 Table — (DOCX) [file pone.0310865.s001.docx]

S1 Table. Instrument gradient elution procedure

| Time  /min | Flow rate /(mL/min) | Mobile phase A/% | Mobile phase B/% |
| --- | --- | --- | --- |
| 0.00 | 0.2 | 95 | 5 |
| 5.00 | 0.2 | 90 | 10 |
| 10.00 | 0.2 | 80 | 20 |
| 20.00 | 0.2 | 75 | 25 |
| 27.00 | 0.2 | 5 | 95 |
| 30.00 | 0.2 | 5 | 95 |
| 30.10 | 0.2 | 95 | 5 |

A : 0.2 % formic acid and 2mM ammonium acetate aqueous solution.

B : LC-MS grade acetonitrile
