# Supplementary material for: Spatial and temporal distribution and ecological risk assessment of typical antibiotics in natural and wastewater of Jinjiang River Basin
Source: PLoS One. 2024 Nov 14;19(11):e0310865. doi: 10.1371/journal.pone.0310865 (PMC11563446; doi:10.1371/journal.pone.0310865)
Supplement: S2 Table — (DOCX) [file pone.0310865.s002.docx]

S2 Table. Mass spectrometry conditions of UPLC-MS /MS for determination of antibiotic

| Species | Compound | Precursor ion  (m/z) | Product ion  (m/z) | Declustering potential/ V | Collision energy /V |
| --- | --- | --- | --- | --- | --- |
| MLs | AZM | 749.2 | 591.1/157.6 | 160 | 32/42 |
|  | RTM | 837.2 | 679.2/157.9 | 175 | 22/36 |
|  | CTM | 748.2 | 158.1/590.5 | 165 | 29/17 |
|  | ERY | 734.1 | 157.9/576.1 | 146 | 30/18 |
|  | ERY-13C,D_3_* | 738.2 | 161.9/580.2 | 162 | 36/20 |
| TCs | TC | 445.1 | 410/427 | 115 | 15/10 |
|  | DOC | 445.1 | 428/97.8 | 135 | 20/45 |
|  | OTC | 461.1 | 425.9/443 | 140 | 19/10 |
|  | CTC | 479.1 | 461.9/444 | 135 | 15/20 |
|  | DTC* | 465 | 447.8/429.8 | 140 | 18/20 |
| SAs | SPD | 250 | 155.8/107.7 | 113 | 14/27 |
|  | SDZ | 251 | 91.9/155.8 | 97 | 25/14 |
|  | SMX | 254.1 | 156.1/108.6 | 100 | 15/25 |
|  | STZ | 256 | 155.9/91.9 | 87 | 14/25 |
|  | SMZ | 279 | 185/92 | 119 | 15/29 |
|  | STP | 281 | 155.8/92 | 133 | 14/25 |
|  | SFM | 281 | 155.9/92 | 107 | 15/27 |
|  | SQX | 300.9 | 155.7/91.9 | 123 | 13/30 |
|  | SMR-D_4_* | 265 | 92/155.9 | 120 | 27/15 |
| FQs | DMZ | 142 | 96/81 | 100 | 15/30 |
|  | MDZ | 172 | 128/82 | 100 | 12/25 |
|  | ENO | 321 | 302.9/231.9 | 130 | 17/37 |
|  | CIP | 340 | 322.1/235 | 150 | 20/47 |
|  | LOM | 352 | 333.9/265 | 130 | 20/22 |
|  | ENR | 360 | 342.1/316.1 | 120 | 20/30 |
|  | OFL | 362 | 318/260.9 | 120 | 17/28 |
|  | FLE | 369.9 | 325.9/268.9 | 140 | 20/30 |
|  | GAT | 375.9 | 357.9/332 | 148 | 18/18 |
|  | NOR | 320.0 | 302.1/276.1 | 130 | 20/15 |
|  | SPA | 393 | 349/291.9 | 160 | 28/28 |
|  | CIP-D8* | 332.0 | 314.1/231.0 | 150 | 18/42 |
|  | Simeton# | 198.1 | 68.2/100.1 | 130 | 35/28 |

Note : * represents the deuterated indicator, # represents the internal standard.
