# Supplementary material for: Spatial and temporal distribution and ecological risk assessment of typical antibiotics in natural and wastewater of Jinjiang River Basin
Source: PLoS One. 2024 Nov 14;19(11):e0310865. doi: 10.1371/journal.pone.0310865 (PMC11563446; doi:10.1371/journal.pone.0310865)
Supplement: S3 Table — (DOCX) [file pone.0310865.s003.docx]

S3 Table. Limit of Detection, Limit of Quantitation, and Standard Curve of Antibiotics

| Category | Compound | Retention time /min | Linear equation | R^2^ | LOD  (ng /L) | LOQ  (ng /L) |
| --- | --- | --- | --- | --- | --- | --- |
| MLs | ERY | 22.800 | y=0.005693*x+6.822883E-004 | 0.99585 | 0.03 | 0.11 |
|  | AZM | 23.781 | y=0.033951*x+0.019734 | 0.99737 | 0.08 | 0.23 |
|  | CTM | 23.782 | y=0.593054*x+0.583071 | 0.99848 | 0.01 | 0.04 |
|  | RTM | 23.875 | y=0.202680*x+0.033912 | 0.99807 | 0.02 | 0.03 |
| TCs | OTC | 10.087 | y=0.023482*x+0.177569 | 0.99768 | 0.06 | 0.17 |
|  | TC | 10.961 | y=0.050848*x+0.096443 | 0.99765 | 0.44 | 1.36 |
|  | CTC | 14.520 | y=0.013177*x+0.027676 | 0.99681 | 0.38 | 1.29 |
|  | DOC | 16.233 | y=0.029338*x-0.094078 | 0.99702 | 0.16 | 0.43 |
| NDs | MDZ | 4.749 | y=0.002988*x+0.005702 | 0.99817 | 0.02 | 0.07 |
|  | DMZ | 5.483 | y=0.003177*x+0.005991 | 0.99951 | 0.04 | 0.11 |
| SAs | SDZ | 6.464 | y=0.013495*x-0.018668 | 0.99790 | 0.09 | 0.27 |
|  | SPD | 8.000 | y=0.012854*x-0.022558 | 0.99565 | 0.01 | 0.02 |
|  | STP | 11.135 | y=0.050943*x+0.030986 | 0.99746 | 0.07 | 0.19 |
|  | STZ | 8.023 | y=0.011060*x-0.008200 | 0.99610 | 0.02 | 0.07 |
|  | SMZ | 10.191 | y=0.022821*x-0.011491 | 0.99584 | 0.10 | 0.31 |
|  | SMX | 14.733 | y=0.011574*x+0.009981 | 0.99915 | 0.10 | 0.21 |
|  | SQX | 20.648 | y=0.006299*x-0.015021 | 0.99584 | 0.21 | 0.67 |
|  | SFM | 11.135 | y=0.050943*x+0.030986 | 0.99746 | 0.01 | 0.02 |
| FQs | ENO | 10.060 | y=0.019191*x-0.051695 | 0.99581 | 0.05 | 0.17 |
|  | FLE | 10.401 | y=0.012103*x-0.025865 | 0.99737 | 0.01 | 0.09 |
|  | NOR | 10.461 | y=0.013639*x-0.003411 | 0.99912 | 0.05 | 0.22 |
|  | OFL | 10.475 | y=0.026106*x-0.019180 | 0.99756 | 0.02 | 0.05 |
|  | CIP | 10.769 | y=0.014527*x-0.028042 | 0.99576 | 0.09 | 0.30 |
|  | LOM | 11.131 | y=0.013231*x+0.017709 | 0.99722 | 0.17 | 0.57 |
|  | ENR | 11.675 | y=0.017753*x-0.009574 | 0.99949 | 0.10 | 0.35 |
|  | GAT | 12.327 | y=0.004165*x+0.031921 | 0.99879 | 0.01 | 0.03 |
|  | SPA | 13.127 | y=0.007398*x+0.013443 | 0.99569 | 0.02 | 0.04 |
