# Supplementary material for: Spatial and temporal distribution and ecological risk assessment of typical antibiotics in natural and wastewater of Jinjiang River Basin
Source: PLoS One. 2024 Nov 14;19(11):e0310865. doi: 10.1371/journal.pone.0310865 (PMC11563446; doi:10.1371/journal.pone.0310865)
Supplement: S4 Table — (DOCX) [file pone.0310865.s004.docx]

S4 Table. Recoveries of antibiotics in spiked surface water and pure water samples (n=3)

| Compound | | Ultrapure water recovery rate % | | | | Surface water recovery rate % | | |
| --- | --- | --- | --- | --- | --- | --- | --- | --- |
|  |  | 10 ng·L^-1^ | 50 ng·L^-1^ | 100 ng·L^-1^ | | 10 ng·L^-1^ | 50 ng·L^-1^ | 100 ng·L^-1^ |
| MLs | ERY | 64.51±3.57 | 58.35±2.42 | 59.88±3 | 73.93±8.32 | | 73.21±2.83 | 73.68±4.01 |
|  | AZM | 85.78±1.52 | 37.54±1.65 | 33.59±4.85 | 280.02±4.38 | | 152.75±9.26 | 144.2±10.62 |
|  | CTM | 40.54±4.17 | 46.69±1.93 | 38.03±1.36 | 26.22±3.4 | | 32.03±1.65 | 30.73±0.22 |
|  | RTM | 50.02±3.4 | 51.07±1.79 | 50.51±0.43 | 58.26±11.96 | | 58.96±0.38 | 57.36±0.84 |
| TCs | OTC | 82.14±4.15 | 85.57±1.94 | 90.13±3.47 | 103.77±10.91 | | 107.32±2.3 | 99.78±3.21 |
|  | TC | 72.75±4.09 | 75.22±1.94 | 83.91±1.25 | 120.36±6.28 | | 104.69±8.67 | 93.03±1.27 |
|  | CTC | 64.7±3.23 | 65.45±3.59 | 86.9±6.96 | 48.15±0.57 | | 61.02±5.25 | 70.4±1.05 |
|  | DOC | 71.7±5.55 | 91.58±7.87 | 109.1±7.55 | 62.98±4.49 | | 56.67±6.04 | 60.85±0.17 |
| NDs | MDZ | 25.38±9.91 | 33.09±2.7 | 28.2±3.72 | 131.38±3.77 | | 93.92±0.77 | 89.93±1.25 |
|  | DMZ | 71.01±10.24 | 80.23±3.37 | 85.59±2.62 | 106.61±11.05 | | 137.57±2.46 | 133.31±1.55 |
| SAs | SDZ | 79.16±8.76 | 83.62±3.58 | 88.72±0.97 | 72.23±0.01 | | 85.16±5.28 | 84.3±2.7 |
|  | SPD | 72.53±8.51 | 82.3±2.5 | 84.8±2.34 | 57.9±0.25 | | 65.71±6.91 | 64.93±1.17 |
|  | STZ | 67.57±8.04 | 81.5±2.35 | 81.37±1.79 | 50.21±1.71 | | 57.89±7.43 | 68.11±1.91 |
|  | SMZ | 67.39±7.98 | 79.95±2.53 | 88.82±2.37 | 63.82±0.94 | | 68.9±4.65 | 68.6±1.77 |
|  | SFM | 64.55±8.62 | 92.11±3.67 | 86.13±4.31 | 52.47±1.25 | | 58.59±6.25 | 63.74±2.51 |
|  | STP | 64.55±8.62 | 84.59±3.67 | 87.99±4.31 | 52.47±1.25 | | 56.16±6.06 | 61.8±2.44 |
|  | SMX | 102.05±3.59 | 112.58±1.05 | 103.1±1.96 | 182.17±4.09 | | 99.61±1.3 | 93.58±3.4 |
|  | SQX | 63.4±7.5 | 76.61±4.52 | 78.17±1.6 | 68.73±1.12 | | 110.79±2.84 | 83.79±2.9 |
| FQs | ENO | 55.18±9.95 | 69.74±6.87 | 83.07±4.82 | 99.64±16.03 | | 112.85±6.18 | 116.15±1.53 |
|  | FLE | 52.37±7.73 | 78.97±4.93 | 87.67±3.7 | 72.62±4.98 | | 85.85±4.53 | 88.67±0.72 |
|  | NOR | 56.41±7.51 | 73.13±6.65 | 86.46±4.39 | 82.26±15.93 | | 128.31±7.53 | 129.9±7.36 |
|  | OFL | 53.87±9.36 | 79.7±7.31 | 80.66±3.83 | 63.35±23.32 | | 90.96±4.82 | 87.12±1.75 |
|  | CIP | 58.48±4.28 | 70.39±7.89 | 80.01±3.43 | 81.38±2.83 | | 85.69±5.58 | 98.31±3.85 |
|  | LOM | 61.3±6.67 | 86.84±5.43 | 88.22±3.58 | 93.86±6.51 | | 97.63±0.49 | 100.18±0.59 |
|  | ENR | 43.99±7.4 | 69.24±7.08 | 81.32±5.1 | 107.51±14.18 | | 139.2±2.8 | 161.36±4.34 |
|  | GAT | 58.98±7.11 | 81.09±7.81 | 87.43±6.19 | 103.7±5.42 | | 100.4±0.35 | 101.96±1.3 |
|  | SPA | 75.88±8.64 | 95.99±8.4 | 80.11±8.37 | 113.62±5.99 | | 100±2.11 | 96.73±3.44 |
